# Resilience and Stress during Pregnancy: A Comprehensive Multidimensional Approach in Maternal and Perinatal Health

**DOI:** 10.1155/2021/9512854

**Published:** 2021-08-13

**Authors:** A. C. Alves, J. G. Cecatti, R. T. Souza

**Affiliations:** Department of Obstetrics and Gynecology, School of Medical Sciences, University of Campinas, Campinas, SP, Brazil

## Abstract

This narrative review addresses resilience and stress during pregnancy, which is part of a broader concept of maternal health. Pregnancy and postpartum are opportune periods for health promotion interventions, especially because the close contact of the women with health professionals. In this way, it can be considered a useful window of opportunity to identify women at higher risk for adverse outcomes. Integrated health is a concept that aims at providing comprehensive care related to the promotion of individuals' physical, mental, and social well-being. In this context, stress during pregnancy has been targeted as a remarkable condition to be addressed whether due to individual issues, social issues, or specific pregnancy issues, since it is directly and indirectly associated with pregnancy complications. Stress is associated with preterm birth, postpartum depression, anxiety, child neurodevelopment, and fetal distress. The way that an individual faces a stressful and adverse situation is called resilience; this reaction is individual, dynamic, and contextual, and it can affect maternal and fetal outcomes. Low resilience has been associated with poorer pregnancy outcomes. The social context of pregnancy can act as a protective or contributory (risk) factor, indicating that environments of high social vulnerability play a negative role in resilience and, consequently, in perceived stress. A given stressor can be enhanced or mitigated depending on the social context that was imposed, as well as it can be interpreted as different degrees of perceived stress and faced with a higher or lower degree of resilience. Understanding these complex mechanisms may be valuable for tackling this matter. Therefore, in the pregnancy-puerperal period, the analysis of the stress-resilience relationship is essential, especially in contexts of greater social vulnerability, and is a health-promoting factor for both the mother and baby.

## 1. Pregnancy and Maternal Health: Remarkable Concepts beyond the Fairy Tale

The broadest concept of health defined by the World Health Organization (WHO) is based not merely on the absence of disease, but in the presence of physical, mental, and social well-being of an individual [1]. Women's reproductive healthcare, including diverse specificities of the pregnancy-puerperal cycle, could be no different. One of the greatest challenges in obstetric healthcare is to assure the quality of prenatal care, improve indicators related to morbidity and mortality due to preventable causes during this period, and also guarantee a positive experience during prenatal care, assuring the promotion and inclusion of social, cultural, emotional, and psychological aspects [2].

At the same time that pregnancy is considered a transitory process, maternity causes definitive modifications in a woman. Changes in a pregnant woman who assumes a maternal role have been studied in the theory elaborated by Ramona Mercer, titled “Attainment of the Maternal Role” [3]. This theory addresses the construction of maternal identity, while redefining a woman's self-perception, and the physical and emotional modifications in her sociocultural dynamics. This interactive evolutionary biopsychosocial complex process between the mother and child, according to the author, consists of four phases. The first is the commitment and preparation phase. It starts in early pregnancy and encompasses social and emotional adaptations inherent in the gestational period [3]. Thus, the Women's Integrated Healthcare National Policy Guidelines of 2004 in Brazil [4] recommends the promotion of qualified humanized obstetric and neonatal care. According to these guidelines, *“Integrated healthcare in women encompasses management of a woman from a broad perception of life context, from the time that she presents a certain demand, as well as her singularity and conditions as an individual capable (of) and responsible for her own choices”* [4].

Humanization in healthcare is a continuous process that demands reflection, since physical and emotional issues are inseparable aspects. Nevertheless, it is worth mentioning that the Health Ministry recommendations for prenatal care are limited to concepts of sickness, risk of complications, and interventions for disease identification or prevention. There is little mention of the importance of the evaluation and management of the emotional demands of the pregnant woman, contrary to the concept of quality of care that should refer to a group of aspects including physical and biopsychosocial issues [4–6].

## 2. Stress and Pregnancy

The term stress is more widely used, despite other meanings such as “tension,” “fatigue,” and “tiredness.” Nevertheless, the term has become popular in colloquial language and in medicine. Nowadays, the concept has other meanings that go beyond these aspects [7–9]. According to Filgueira and Hippert, “stress” is a state manifested by a specific syndrome, consisting of all nonspecific alterations produced in a biological system. According to those authors, stress (physical, psychological, or social) may be understood as a term encompassing a group of reactions and stimuli that cause disturbances in the body equilibrium, frequently with damaging effects [7].

Stress can be defined, therefore, as a natural reaction of an organism to adverse situations that disturb its homeostasis or balance. The body responds in a state of alert, implying different physical and emotional alterations, which generate different degrees of adaptation to the causative agent. These agents may be acute or chronic and result from the external environment. Interpersonal, family, and work may be involved, in addition to physical injuries, diseases, and others. This means that these agents may result from the environment where the individual is inserted. They may also derive from internal factors, related to exhaustion, tension, and other emotional factors [8, 9].

In a lower or higher intensity, pregnancy is a period of emotional alterations, resulting from both social and psychological factors, as well as typical hormonal alterations [10, 11]. Some stressors are related to both specific events and physiological adaptations expected in the maternal body: nausea, weight gain, insomnia, and emotional lability. Individual factors, such as unplanned pregnancies, changes in family dynamics such as the relationship with a partner, acquired responsibilities with neonatal care, and the risk of complications during pregnancy and labor are other stressors [10–12]. Another important factor which can be an aggravating stressor for pregnant women is the socioeconomic context: low income, domestic violence, use of drugs and alcohol, lack of a family support network, and other vulnerabilities [13].

In a study of 2010 including more than 1,500 women, stress was evaluated by the Prenatal Psychosocial Profile stress scale. Research results show that 6% (*n* = 91) of the women were classified as having a high level of stress, the large part of these pregnant women, 78% (*n* = 1.190) reported low or moderate stress, and only 16% (*n* = 241) demonstrated no stress [14].

Some studies show that this exposure during pregnancy, mainly if persistent or long term, may be related to adverse maternal and perinatal outcomes. In the last decades, various studies have demonstrated that stress in pregnancy may predispose to preterm labor (before 37 weeks) and pregnancies resulting in small for gestational age newborns (less than the 10^th^ percentile of the expected weight for gestational age). [15–19] The literature also indicates that there is a higher incidence of psychiatric disturbances in a woman during pregnancy and the postpartum period. Adequate care and follow-up are required for timely detection and opportune intervention [20, 21]. Knowing the perceptions and experiences of a woman related to stress experienced in the pregnancy-puerperal cycle may favor a healthy labor and postpartum period and is an opportunity to welcome and support women, families, and community as a whole [6–14]. A research from 2017 described an association between the number of stressor events during pregnancy and the impact on the pregnant woman, with the occurrence of postpartum depression and other common mental disorders in pregnancy, including anxiety and insomnia [22]. Primipara, who are going through the experience of pregnancy for the first time, deserve special care, since the unprecedented physiological and psychological changes in the gestational period, as well as transition to the social maternal role, may by itself represent a stressor factor. It is important to identify pregnant women or groups at risk for stress and anxiety, to prevent adverse outcomes in maternal and perinatal healthcare [14].

Nevertheless, it is known that perception of a stressor factor is individual and dependent on the personal capacity to elaborate. A woman may or not have significant stress symptoms in the presence of a stressor factor. According to Cohen and Williamson [23, 24], there is more than one way to measure stress. Specific stressor agents may, for example, be demonstrated, quantified, and qualified. Physical and psychological symptoms originating from exposure to stress may be identified. Finally, the individual perception of stress, irrespective of triggering stressors, may be measured. Researchers have developed a perceived stress scale, aimed at measuring individual perception of subjects exposed to stressful situations. This scale was named the Perceived Stress Scale (PSS) and had 14 items (PSS 14) [23], but was later validated with ten items (PSS 10) [23–25] and even more briefly in another version with four questions (PSS 4) [24, 25]. PSS 4 has been especially used during situations where there is a short time to measure the perception of stress, as in telephone surveys. According to the authors, the items were developed to identify how much individuals considered their lives unpredictable and uncontrollable and how much they felt overwhelmed [24, 25]. These parameters have been considered fundamental in the individual's perception of stress. An advantage of PSS is the lack of specific context questions, which makes its transcultural validation, as well as its applicability and demographic contexts possible [26–28]. After all, the same context and/or stressor factor may be perceived in different degrees by each individual, generating distinct consequences and outcomes, increasing the importance of this evaluation [23–25].

A more recent approach in stress measurement during pregnancy focuses on pregnancy-specific stress, i.e., conditions directly related to pregnancy that increase a woman's level of stress [29–31]. Among these conditions, we could include body changes and pregnancy-related adaptations, pregnancy-specific symptoms, in addition to concerns and tensions inherent to maternity, and the new social relationship that is constructed with the pregnancy [30, 31]. Study results by Lobel et al. from 2008 indicated that pregnancy-specific stress may be the best predictor of adverse perinatal outcomes rather than the evaluation of general stress factors, such as the degree of anxiety or stress perceived in general [29]. Pregnancy-specific stress was associated with preterm labor and unhealthy habits in relation to feeding, physical activity, and smoking. The latter was related to low birth weight. By association, the pregnancy-specific stress would be indirectly related to this adverse outcome [29].

Maternal stress may be related not only to short-term perinatal outcomes. In the newborn, the consequences may be seen in the late neonatal life or infancy. There is evidence that stress, depression, and anxiety during pregnancy are related to neurodevelopmental effects on infants, including lower cephalic circumference, worse cognitive development, and behavioral disturbances in infancy [32]. A prospective study investigated stress during pregnancy in a sample of 170 nulliparous and followed the development of newborns at 3 and 8 months. The results demonstrated a higher rate of delay in motor and mental development in children whose mothers demonstrated higher stress levels during pregnancy [33].

Furthermore, a recent study demonstrated that gestational stress may even interfere in fetal longevity. Send et al. studied fetal and maternal telomeres and considered biomarkers of aging [34]. Research results took into consideration a telomere length of 319 newborns and 318 mothers and demonstrated that perceived stress during pregnancy was associated with shorter telomeres in newborn infants, but there was no relationship with maternal telomere length. This demonstrates that fetal development is probably vulnerable to the exposure to stress [34].

A study of 227 Chinese pregnant women showed an association between perceived stress and quality of sleep during pregnancy, demonstrating that higher levels of stress were negatively associated with the quality of sleep in these pregnant women. Furthermore, it showed that higher levels of resilience were significantly associated with a better quality of sleep and were considered protective factors. Resilience had a mediating role between maternal stress and quality of sleep (*p* < 0.01) [35].

It is perceived that the ability to deal with stressful situations is also determined by a series of complex genetic mechanisms that are strongly influenced by individual factors, sex, age, and temperament, as well as by social environmental action [36–38].

## 3. Resilience: Human Capacity between Stress and “Well-Being”

Psychology has studied the individual human reaction to adverse circumstances and/or stressor factors, termed resilience. This reaction is independent of the intensity or quality of the stressors. It considers individual response and coping mechanisms that should be analyzed in a specific context in the face of an expected response (for example, same age group and sociocultural context) [39].

Resilience may be defined as the capacity to adapt to life adversities and is considered a subjective measure of this response that encompasses concepts such as inner strength, competence, and flexibility. It may be inversely related to depression, perception of stress, and anxiety [40]. This is a dynamic characteristic, as studies have shown in the evaluation of elderly adults. Some authors suggest that resilience may increase during adult life, probably due to a positive effect of overcoming limits and adversities during life [40, 41]. At the same time, it is not necessarily an increasingly constant attribute, but rather a relative adaptable behavior, according to individual circumstances and contexts. People that deal successfully with stress and adversity during a certain period of life may react adversely in other situations and other time periods [39–41].

The bibliographic review of instruments for the evaluation of resilience in the Brazilian context showed that there is still a lack of instruments for a direct evaluation of this characteristic. A large part of the constructs approved for use indirectly evaluate resilience through risk factors and protection related to the concept: personality, psychopathologies (especially stress and anxiety), family history, and environmental/social factors [42]. Only two scales meet these characteristics: the Wagnild and Young and Connor–Davidson Resilience Scale [43, 44]. Both were validated and translated to Portuguese with the original reduced versions available, as explained below.

The Wagnild and Young Resilience Scale from 1993 is one of the most widely used instruments in the evaluation of resilience [43, 45]. Its transcultural adaptation to Portuguese was presented by Pesce et al. According to them, Cronbach's alpha scores, a coefficient that measures the reliability of questions contained in a certain assessment instrument of the Brazilian version, are similar to those reported by Wagnild and Young in 1993, demonstrating satisfactory internal consistency of the adapted scale (Alfa de Cronbach: 0.80) [46].

The instrument consists of 25 items, each scored from 0 to 7, according to the Likert scale, varying in “agree” (subclassified as weakly, strongly, or totally), “I neither agree, nor disagree,” and “disagree” (weakly, strongly, or totally). It was proposed that the level of agreement is the degree of concordance among items that reflect the theoretical definition of resilience. It is composed of two factors, as established by the original study (Wagnild and Young). Factor I : personal competence, which indicates self-confidence, independence, decision, invincibility, power, ingenuity, and perseverance. Factor II : acceptance of self and life, which represents the capacity to adapt, balance, be flexible, and have a stable life perspective that coincides with accepting life with serenity, despite the adversities [43, 45]. The Resilience Scale has a reduced and validated version of 14 items (RS-14), a version published in 2009 by Gail Wagnild, one of the authors of the original scale, and a good level of reliability was maintained [47].

For the evaluation of resilience in studies addressing the subject, another commonly used instrument is the Connor–Davidson Resilience Scale (CD-RISC). The instrument was developed by Connor and Davidson in 2003 and revalidated by Campbell-Sills and Stein in 2007 [44, 48]. The original Connor and Davidson Scale has 25 items. However, in confirmatory factor analysis, Campbell-Sills and Stein identified a 10-item version that was renamed CD-RISC-10, differentiating it from the original form [48]. CD-RISC-10 was validated into the Brazilian context by Lopes and Martins in 2010 [49].

In a case-control study in 2010, Salazar-Pousada et al. analyzed 302 pregnant women, comparing differences between resilience and depressive symptoms in groups of adolescents and adults. In that analysis, the 14-item Wagnild and Young resilience Scale was applied. The adolescent group had lower scores (less resilience) and higher scores that were lower than the median calculated in the sample (*p* < 0.05). Having an adolescent and preterm birth were factors related to a higher risk of low resilience (OR, 3.0 95%CI 1.43–6.55, *p*=0.004) [50].

The relationship between resilience and mood disorders has been investigated in pregnant women. Some studies showed that individuals with high levels of resilience tend to have less symptoms of depression and are more emotionally balanced [51, 52]. Therefore, in pregnancy, a time of important psychosocial adaptation, a high level of resilience would be important to adapt to changes inherent to the gestational period and maternity. A study of 531 pregnant women indicated that those with high trait anger were more inclined to have lower levels of resilience, which probably is related to the development of higher rates of postpartum depression in this group [53].

Psychobiology, also known as behavioral neuroscience, offers a possible explanation for the association between resilience and mood disorders. Physiologically, an organism undergoing stressful situations releases corticotrophin-releasing hormone (CRH) by the hypothalamus, activating the hypothalamic-pituitary-adrenal axis (HPA), ultimately leading to cortisol release by the adrenal glands. The defense response to stress (fight or flight) is related to autonomic, cognitive, emotional, and behavioral alterations in normal conditions. In the short term, cortisol has a protective action and enables an adequate response to the situation, whether it is a physical or emotional stressor, and cortisol levels return to baseline values after stimulus cessation. Nevertheless, sustained exposure to abnormally increased cortisol levels may be damaging and result in hypertension, immunosuppression, cardiovascular disease, and other health problems. Neuroscience attempts to establish the biological role of resilience in this chain, associating higher levels of resilience with individual capacity of a complex negative feedback system that balances glucocorticoid and mineralocorticoid receptors at an optimal level of response. It is believed that resilience would cause the hypothalamic-pituitary-adrenal (HPA) axis to reach an ideal activation level, so that it responds to a stressor factor, as required, but does not exacerbate reactions such as anxiety, excessive fear, and depression [54].

The evaluation of the level of resilience in pregnant women may facilitate coping with difficulties inherent to the period, such as fears related to body changes and adaptations, as well as fears related to labor and social problems, among other reasons in each pregnant woman [55]. Identifying groups with a lower level of resilience may help detect individuals who are at a higher risk and have less access to resources required to face pregnancy-specific difficulties. This may contribute to individual care of each pregnant woman and target intervention strategies in conformity [55, 56].

The concept of social vulnerability may be applied to individuals experiencing adversities in their daily living; i.e., it may be associated with risk factors that negatively affect the social reality of this individual. It is characterized as an unfavorable situation, in comparison to other population groups. The more the risk factors composing this reality, the lower the protection, the greater the vulnerability, and thus, the higher the probability of adverse consequences for psychosocial development [57–59]. Risk factors are considered behaviors or conditions that are damaging to an individual's health and well-being, as well as the lack of protective or attenuating factors in the social context. Diverse factors are highlighted: socioeconomic, environmental, and demographic conditions, social relationships, and subjectivity [60].

The distribution of vulnerability factors in pregnant women is not homogeneous. There may be an accumulation in some of these women, who would be exposed to a greater risk of adverse maternal and perinatal results. An early identification of these vulnerability factors may aid in management and promote a subjective and individual action in women that have a higher exposure to risk. In addition to providing the formulation of proper public policies and programs in the promotion of individual and collective health, this approach may substantiate the identification of more resilient pregnant women, modulating the perception of stress and coping skills. As a result, morbidity and mortality could decrease and gestational health would be addressed in a broad integrated manner [61].

In a prospective cohort study from 2016, Maxson et al. analyzed gestational outcomes in an approach termed psychosocial health profiles. Women were grouped together into clusters and classified as resilient, moderate, and vulnerable. The vulnerable profile grouped pregnant women with a higher level of perceived stress and depression, lower self-confidence, less paternal support, and lower interpersonal support network. Women also differed in sociodemographic characteristics: these women tended to be younger, had lower level of schooling, and were not in a stable relationship. Women in the resilient group had lower rates of premature delivery than women in the other two groups. Of the 1313 women analyzed, 186 (14.1%) had premature deliveries (before 37 weeks), and the rates were 11% in the resilient group and 16.2% in the other two groups. Comparatively, the resilient group had a 52% lower rate of preterm delivery compared to the vulnerable group and a 40% lower rate compared to the moderate (adjusted OR and 95% CI). In addition to higher preterm birth rates, this group also had higher rates of unplanned and unwanted pregnancy. Multidimensional analysis of health in pregnancy helps in the identification of this vulnerability profile and is an important window of opportunity for interventions that decrease risks and consequences, since prenatal care is a singular time when healthcare provides regular access to these women [62].

## 4. Final Considerations

In the presence of one or more stressor factors, a woman will have her individual perception and face adversities according to her resilience. It is known that resilience varies, depending on personal characteristics and the context in which the woman is inserted [53, 55]. In a more encompassing view of integrated healthcare, it is equally important to evaluate the sociodemographic contexts and individual aspects permeating stress and resilience in a woman during pregnancy. Although the social environment is a source of stress [63], it may also be a protective factor in crisis interventions, since social support may aid in coping. In the same manner, the lack of a favorable context may act as a vulnerability factor for the pregnant woman [62]. There are tools available for stress and resilience assessments that may be applied during pregnancy and can help in the multidimensional evaluation of maternal health. The aim of this proposal was to obtain a broader view of subjectivity in maternal health, considering disease prevention, seeking the promotion of a positive maternity experience as early as prenatal care [2, 5].

## References

[1] WHO *Frequently asked questions – World Health Organization*.

[2] WHO (2016). *WHO Recommendations on Antenatal Care for a Positive Pregnancy Experience*.

[3] Mercer R. T. (2004). Becoming a mother versus maternal role attainment. *Journal of Nursing Scholarship*.

[4] Política nacional de atenção integral à saúde da mulher: princípios e diretrizes (2004). *Princípios e diretrizes*.

[5] Programa de Humanização no Pré-Natal e Nascimento (2001). *Informações para gestores e técnicos*.

[6] Ministério da Saúde (2006). *Pré-Natal e Puerpério—atenção qualificada e humanizada*.

[7] Figuerias J. C., Hippert M. I. S. (1999). A polêmica em torno do conceito de estresse. *Psicologia: Ciência e Profissão*.

[8] Chrousos G. P., Gold P. W. (1992). The concepts of stress and stress system disorders. Overview of physical and behavioral homeostasis. *Journal of the American Medical Association*.

[9] Margis R., Picon P., Cosner A. F., Silveira R. D. O. (2003). Relação entre estressores, estresse e ansiedade. *Revista de Psiquiatria do Rio Grande do Sul*.

[10] Gonzalez-Ochoa R., Sánchez-Rodríguez E. N., Chavarría A., Gutiérrez-Ospina G., Romo-González T. (2018). Evaluating stress during pregnancy: do we have the right conceptions and the correct tools to assess it?. *Journal of Pregnancy*.

[11] Coutinho E. D. C., Silva C. B. D., Chaves C. M. B. (2014). Pregnancy and childbirth: what changes in the lifestyle of women who become mothers?. *Revista da Escola de Enfermagem da USP*.

[12] Ruiz R., Fullerton J. (1999). The measurement of stress in pregnancy. *Nursing and Health Sciences*.

[13] Sousa W. P. D. S. (2015). *Resiliência e apoio social em gestantes tardias*.

[14] Woods S. M., Melville J. L., Guo Y., Fan M. Y., Gavin A. (2010). Psychosocial stress during pregnancy. *American Journal of Obstetrics and Gynecology*.

[15] Copper R. L., Goldenberg R. L., Das A. (1996). The preterm prediction study: maternal stress is associated with spontaneous preterm birth at less than thirty-five weeks’ gestation. National Institute of Child Health and Human Development Maternal-Fetal Medicine Units Network. *American Journal of Obstetrics and Gynecology*.

[16] Dole N., Savitz D. A., Hertz-Picciotto I., Siega-Riz A. M., McMahon M. J., Buekens P (2003). Maternal stress and preterm birth. *American Journal of Epidemiology*.

[17] Hedegaard M., Henriksen T. B., Secher N. J., Hatch M. C., Sabroe S. (1996). Do stressful life events affect duration of gestation and risk of preterm delivery?. *Epidemiology*.

[18] Davis E. P., Glynn L. M., Waffarn F., Sandman C. A (2011). Prenatal maternal stress programs infant stress regulation. *The Journal of Child Psychology and Psychiatry and Allied Disciplines*.

[19] Hoffman S., Hatch M. C. (1996). Stress, social support and pregnancy outcome: a reassessment based on recent research. *Paediatric and Perinatal Epidemiology*.

[20] Bernazzani O., Saucier J.-F., David H., Borgeat F. (1997). Psychosocial factors related to emotional disturbances during pregnancy. *Journal of Psychosomatic Research*.

[21] Pais M., Pai M. V. (2018). Stress among pregnant women: a systematic review. *Journal of Clinical and Diagnostic Research*.

[22] Alvarenga P., Frizzo G. B. (2017). Stressful life events and women’s mental health during pregnancy and postpartum period. *Paideia*.

[23] Cohen S., Kamarck T., Mermelstein R. (1983). A global measure of perceived stress. *Journal of Health and Social Behavior*.

[24] Cohen S., Spacapan S., Oskamp S. (1988). Perceived stress in a probability sample of the United States. *The Claremont Symposium on Applied Social Psychology—the Social Psychology of Health*.

[25] Lee E. H. (2012). Review of the psychometric evidence of the perceived stress scale. *Asian Nursing Research*.

[26] Lesage F. X., Berjot S., Deschamps F. (2012). Psychometric properties of the French versions of the perceived stress scale. *International Journal of Occupational Medicine and Environmental Health*.

[27] Leung D. Y., Lam T. H., Chan S. S. (2010). Three versions of Perceived Stress Scale: validation in a sample of Chinese cardiac patients who smoke. *BMC Public Health*.

[28] Baik S. H., Fox R. S., Mills S. D. (2019). Reliability and validity of the perceived stress scale-10 in hispanic Americans with English or Spanish language preference. *Journal of Health Psychology*.

[29] Lobel M., Cannella D. L., Graham J. E., DeVincent C., Schneider J., Meyer B. A. (2008). Pregnancy-specific stress, prenatal health behaviors, and birth outcomes. *Health Psychology: Official Journal of the Division of Health Psychology*.

[30] Dipietro J. A., Ghera M. M., Costigan K., Hawkins M. (2004). Measuring the ups and downs of pregnancy stress. *Journal of Psychosomatic Obstetrics and Gynaecology*.

[31] Alderdice F., Lynn F., Lobel M. (2012). A review and psychometric evaluation of pregnancy-specific stress measures. *Journal of Psychosomatic Obstetrics and Gynecology*.

[32] O’donnell K., O’connor T. G., Glover V. (2009). Prenatal stress and neurodevelopment of the child: focus on the HPA axis and role of the placenta. *Developmental Neuroscience*.

[33] Huizink A. C., Robles de Medina P. G., Mulder E. J. H., Visser G. H. A., Buitelaar J. K. (2003). Stress during pregnancy is associated with developmental outcome in infancy. *Journal of Child Psychology and Psychiatry*.

[34] Send T. S., Gilles M., Codd V. (2021). Telomere length in newborns is related to maternal stress during pregnancy. *PsycNET. Neuropsychopharmacology*.

[35] Li G., Kong L., Zhou H., Kang X., Fang Y., Li P. (2016). Relationship between prenatal maternal stress and sleep quality in Chinese pregnant women: the mediation effect of resilience. *Sleep Medicine*.

[36] Sapolsky R. M. (2004). *Why Zebras Don’t Get Ulcers. 3*.

[37] Sadir M. A., Bignotto M. M., Lipp M. E. N. (2010). Stress e qualidade de vida: influência de algumas variáveis pessoais. *Paideia*.

[38] Faro A., Pereira M. E. (2013). Medidas do estresse: UMA revisão narrativa. *Psicologia, Saúde e Doenças*.

[39] Kocalevent R.-D. (2015). Resilience in the general population: standardization of the resilience scale (RS-11). https://journals.plos.org/plosone/article?id=10.1371/journal.pone.0140322.

[40] Wagnild G. M., Collins J. A. (2009). Assessing resilience. *Journal of Psychosocial Nursing and Mental Health Services*.

[41] Bauman S., Adams J. H., Waldo M. (2001). Resilience in the oldest-old - ProQuest. *Counseling and Human Development*.

[42] Knorst C. E. K. (2012). Trabalho de conclusão de especialização (Especialização). *Resiliência: instrumentos de avaliação no contexto brasileiro*.

[43] Wagnild G. M., Young H. M. (1993). Development and psychometric evaluation of the resilience scale. *Journal of Nursing Measurement*.

[44] Connor K. M., Davison J. R. T. (2003). Development of a new resilience scale: the connor-davidson resilience scale (CD-RISC). *Depression and Anxiety*.

[45] Wagnild G. (2009). *Resilience Scale—A Reliable and Valid Tool to Measure Resilience*.

[46] Pesce R. P., Assis S. G., Avanci J. Q., Santos N. C., Malaquias J. V., Carvalhaes R. (2005). Adaptação transcultural, confiabilidade e validade da escala de resiliência. *Cadernos de Saúde Pública*.

[47] Wagnild G. (2009). *The Resilience Scale User’s Guide for the US English Version of the Resilience Scale and the 14-Item Resilience Scale (RS-14)*.

[48] Campbell-Sills L., Stein M. B. (2007). Psychometric analysis and refinement of the connor-davidson resilience scale (CD-RISC): validation of a 10-item measure of resilience. *Journal of Traumatic Stress*.

[49] Lopes V. R., Martins M. D. C. F. (2011). Validação fatorial da escala de resiliência de connor-davidson (CD-RISC-10) para brasileiros. *Revista Psicologia: Organizações e Trabalho*.

[50] Salazar D. P., Arroyo D., Hidalgo L., Pérez-López F. R., Chedraui P. (2011). Depressive symptoms and resilience among pregnant adolescents: a case-control study. *Obstetrics and Gynecology International*.

[51] Edward K.-L. (2005). Resilience: a protector from depression. *Journal of the American Psychiatric Nurses Association*.

[52] Ziaian T., de Anstiss H., Antoniou G., Baghurst P., Sawyer M. (2012). Resilience and its association with depression, emotional and behavioural problems, and mental health service utilisation among refugee adolescents living in South Australia. *International Journal of Population Research*.

[53] Tobe H., Kita S., Hayashi M., Umeshita K., Kamibeppu K. (2020). Mediating effect of resilience during pregnancy on the association between maternal trait anger and postnatal depression. *Comprehensive Psychiatry*.

[54] Feder A., Nestler E. J., Charney D. S., Feder A., Nestler E. J., Charney D. S. (2009). Psychobiology and molecular genetics of resilience. *Nature Reviews Neuroscience*.

[55] García-León M. Á., Caparrós-González R. A., Romero-González B., González-Perez R., Peralta-Ramírez I. (2019). Resilience as a protective factor in pregnancy and puerperium: its relationship with the psychological state, and with Hair Cortisol Concentrations. *Midwifery*.

[56] Pfeiffer C., Ahorlu C. K., Alba S., Obrist B. (2017). Understanding resilience of female adolescents towards teenage pregnancy: a cross-sectional survey in Dar es Salaam, Tanzania. *Reproductive Health*.

[57] Masten A. S., Garmezy N., Lahey B. B., Kazdin A. E. (1985). Risk, vulnerability, and protective factors in developmental psychopathology. *Advances in Clinical Child Psychology*.

[58] Neggers Y., Goldenberg R., Cliver S., Hauth J. (2006). The relationship between psychosocial profile, health practices, and pregnancy outcomes. *Acta Obstetricia et Gynecologica Scandinavica*.

[59] Deems N. P., Leuner B. (2020). Pregnancy, postpartum and parity: resilience and vulnerability in brain health and disease. *Frontiers in Neuroendocrinology*.

[60] Briscoe L., Lavender T., Mcgowan L. (2016). A concept analysis of women’s vulnerability during pregnancy, birth and the postnatal period. *Journal of Advanced Nursing*.

[61] Colciago E., Merazzi B., Panzeri M., Fumagalli S., Nespoli A. (2020). Women’s vulnerability within the childbearing continuum: a scoping review. *European journal of midwifery*.

[62] Maxson P. J., Edwards S. E., Valentiner E. M., Miranda M. L. (2016). A multidimensional approach to characterizing psychosocial health during pregnancy. *Maternal and Child Health Journal*.

[63] Galvão-Coelho N. L., Silver H. P. A., Sousa M. B. C. D. (2015). Resposta ao estresse: II. Resiliência e vulnerabilidade. *Estudos de Psicologia*.

